# Gender-Specific Associations Between Saliva Microbiota and Body Size

**DOI:** 10.3389/fmicb.2019.00767

**Published:** 2019-04-10

**Authors:** Sajan C. Raju, Sonja Lagström, Pekka Ellonen, Willem M. de Vos, Johan G. Eriksson, Elisabete Weiderpass, Trine B. Rounge

**Affiliations:** ^1^Folkhälsan Research Center, Helsinki, Finland; ^2^Faculty of Medicine, University of Helsinki, Helsinki, Finland; ^3^Institute for Molecular Medicine Finland, University of Helsinki, Helsinki, Finland; ^4^Human Microbiome Research, Faculty of Medicine, University of Helsinki, Helsinki, Finland; ^5^Laboratory of Microbiology, Wageningen University, Wageningen, Netherlands; ^6^Department of General Practice and Primary Health Care, University of Helsinki and Helsinki University Hospital, Helsinki, Finland; ^7^Department of Chronic Disease Prevention, National Institute for Health and Welfare, Helsinki, Finland; ^8^Department of Research, Cancer Registry of Norway, Institute of Population-based Cancer Research, Oslo, Norway; ^9^Department of Medical Epidemiology and Biostatistics, Karolinska Institutet, Stockholm, Sweden; ^10^Department of Community Medicine, Faculty of Health Sciences, University of Tromsø, The Arctic University of Norway, Tromsø, Norway

**Keywords:** saliva, microbiota, body mass index, obesity, children

## Abstract

**Objective:**

The human intestinal microbiota likely play an important role in the development of overweight and obesity. However, the associations between saliva microbiota and body mass index (BMI) have been sparsely studied. The aim of this study was to identify the associations between saliva microbiota and body size in Finnish children.

**Methods:**

The saliva microbiota of 900 Finnish children, aged 11–14 years with measured height and weight, was characterized using 16S rRNA (V3–V4) sequencing.

**Results:**

The core saliva microbiota consisted of 14 genera that were present in more than 95% of the Finnish children. The saliva microbiota profiles were gender-specific with higher alpha-diversity in boys than girls and significant differences between the genders in community composition and abundances. Alpha-diversity differed between normal weight and overweight girls and between normal weight and obese boys. The composition was dissimilar between normal weight and obese girls, but not in boys. The relative abundance profiles differed according to body size. Decrease in commensal saliva bacteria were observed in all the body sizes when compared to normal weight children. Notably, the relative abundance of bacteria related to, *Veillonella*, *Prevotella*, *Selenomonas*, and *Streptococcus* was reduced in obese children.

**Conclusion:**

Saliva microbiota diversity and composition were significantly associated with body size and gender in Finnish children. Body size–specific saliva microbiota profiles open new avenues for studying the potential roles of microbiota in weight development and management.

## Introduction

The composition and diversity of the human microbiota may be an important factor in health and disease (Cho and Blaser, 2012). Several studies have proposed that changes in the human gut microbiota may alter pathogenic mechanisms, which are associated with the development of obesity and insulin resistance (Musso et al., 2010). Studies have shown associations between the gut microbiota and body size (Turnbaugh et al., 2006), however, little is known about the saliva microbiota and body size. While most of the microbiota studies have focused on the intestinal microbiota, it is known that the oral cavity possesses the second most diverse microbial community in the body (Kilian et al., 2016). The oral cavity is the major gateway for microorganisms into the human body. Microorganisms enter the body along with food and air through the mouth and are then either mixed with saliva on the way to the intestinal tract, transported to the trachea and lungs or left behind to colonize the oral cavity (Dewhirst et al., 2010). Microorganisms in the oral cavity have a substantial possibility of transmission to contiguous epithelial surfaces nearby and throughout the body (Dewhirst et al., 2010). The oral cavity harbors around 600–700 common species, however, only around 68% of them can be cultivated using anaerobic microbiological methods (Chen et al., 2010; Dewhirst et al., 2010).

The salivary microbiota is more resilient than the facal microbial community toward exposure to antibiotics, irrespective of the type of antibiotics taken (Zaura et al., 2015), and short-term hospitalization does not alter the structure of the saliva microbiome (Cabral et al., 2017). The saliva microbiota exhibits temporal stability and shows little or no evidence of diurnal variation, indicating that the time of the saliva sampling is not critical (Cameron et al., 2015; Belstrøm et al., 2016). Stability of the oral microbiota seems to be high over several years, even when changes in diet and oral hygiene may occur (Stahringer et al., 2012). Oral bacteria have been reported to be associated with a number of oral diseases (Krishnan et al., 2017), but have also been linked to many systemic diseases (Krishnan et al., 2017) and cancer (Mager et al., 2005). Studies have shown that eight bacterial genera (*Streptococcus, Veillonella, Gemella, Granulicatella, Neisseria, Prevotella, Rothia*, and *Fusobacterium*) represent the “core saliva microbiome,” and were observed in >95% of samples (Stahringer et al., 2012).

Previous studies on the oral microbiota and body size in adults show contradicting results. In a Danish cohort study on 292 adults, no association was observed between body mass index (BMI) and the saliva microbiota (Belstrøm et al., 2014). However, studies on microbiota in the oral subgingival biofilm showed a six-fold increase of *Campylobacter rectus* and *Neisseria mucosa* in obese adolescents (*N* = 87) (Zeigler et al., 2012). An Italian study on saliva microbiota of 28 obese and 28 normal weight adults indicated that Firmicutes and Actinobacteria were more abundant in obese individuals, while Proteobacteria and Fusobacteria dominated in normal weight individuals (Piombino et al., 2014). The saliva microbiota has been associated with oral and general health status (Krishnan et al., 2017). A relative increase in *Tannerella forsythia* with increasing BMI has been shown in periodontally healthy adults (Haffajee and Socransky, 2009; Stahringer et al., 2012). Moreover, a recent Danish study on 322 adults reported that the absence or a low count of *Lactobacillus* in the oral cavity may potentially be a novel marker to identify those at increased risk of weight gain (Rosing et al., 2017).

Many studies have investigated the gut microbiota – body size relationship (Graham et al., 2015). However, only a few studies have investigated the association between BMI and the saliva microbiota in children, and most studies have small sample sizes. A recent longitudinal twin study with 107 subjects showed that microbial populations of twins resemble each other more closely when they are young and cohabit (Stahringer et al., 2012). The above-mentioned twin study concluded that genetic and environmental factors influenced the oral microbial composition, and that there was no association with weight, gender, or food preferences (Stahringer et al., 2012). Furthermore, a study on closely related individuals observed that the shared environment had a greater impact on shaping the salivary microbiome than genetics (Shaw et al., 2017).

So far, there are no large-scale studies on the saliva microbiota and body size in children using a culture-independent approach. We aimed to analyze the composition and diversity of the saliva microbiota and to study the associations with body size in a large-scale study of Finnish children. We hypothesize that the saliva microbiota may differ with weight status, and that the observed associations can guide future intervention studies on weight development and management in children.

## Materials and Methods

### Study Population, Design and Samples

One thousand participants aged 11–14 year-old were randomly selected from the prospective Finnish Health in Teens study cohort – Fin-HIT^1^ (Figueiredo et al., 2018). This cohort consists of approximately 11,000 9–14-years-old children and 6500 of their mothers (for some children, fathers, or other legal guardians). The Fin-HIT participants answered a baseline questionnaire, provided a saliva sample (unstimulated), and had their height, weight and waist circumference measured in a standardized manner at school. All participants and one responsible adult for each child provided written informed consent. The regional Ethics Committee of the Hospital, District of Helsinki-Uusimaa region (169/13/03/00/10) approved the study. We randomly selected 1000 saliva samples from the cohort in order to get an unbiased representation of the population. Among the 1000 participants, 25 withdrew their consent after enrolment and their samples were excluded. BMI was calculated (kg/m^2^) using the anthropometric measurements gathered by fieldworkers (Sarkkola et al., 2016), and the participants were classified into four groups according to standard BMI cut-offs for children (Cole and Lobstein, 2012). Samples without sufficient information to calculate BMI and participants using antibiotics 3 months prior to sampling were excluded in the analysis, leaving 900 children (483 boys and 417 girls) to be analyzed (Table 1).

**Table 1:** Body mass index (BMI) category by gender among 900 children aged 11–14 years.

Body size	Boys		Girls		Total	
	*N*	%	*N*	%	*N*	%
Underweight	48	11.5	77	15.9	125	13.9
Normal weight	307	73.6	353	73.1	660	73.3
Overweight	52	12.5	44	9.1	96	10.7
Obese	10	2.4	9	1.9	19	2.1
Total	417		483		900	


*The distribution of the participants according to body sizes and gender are shown in proportion and total number per category after removing individuals without metadata or consent or those with recent antibiotic use.*

The saliva samples were collected in Oragene-DNA (OG-500) self-collection kits (DNA Genotek Inc., Canada). Participants mixed the saliva specimens with a stabilizing reagent within the collection tubes per manufacturer’s instructions, and the tubes were stored at room temperature. A protocol that contained an intensive lysis step using a cocktail of lysozyme and mechanical disruption of bacterial cells using bead-beating was then employed. 50 ml lysozyme (10 mg/ml, Sigma-Aldrich), 6 ml mutanolysin (25 KU/ml, Sigma-Aldrich), and 3 ml lysostaphin (4000 U/ml, Sigma-Aldrich) were added to a 500 ml aliquot of cell suspension, followed by incubation for 1 h at 37°C. Second, 600 mg of 0.1 mm diameter zirconia/silica beads (BioSpec, Bartlesville, OK, United States) were added to the lysate, and the microbial cells were mechanically disrupted using Mini-BeadBeater-96 (BioSpec, Bartlesville, OK, United States) at 2100 rpm for 1 min (Yuan et al., 2012). After lysis, total DNA was extracted using the cmg-1035 saliva kit and Chemagic MSM1 nucleic acid extraction robot (PerkinElmer).

### Amplification and Sequencing

Sample amplification and sequencing libraries were prepared according to an in-house 16S rRNA PCR amplification protocol, with characterized reproducibility and contamination levels (Raju et al., 2018). 16S primers S-D-Bact-0341-b-S-17 (5′ CCTACGGGNGGCWGCAG ′3) and S-D-Bact-0785-a-A-21 (5′ GACTACHVGGGTATCTAATCC 3′) were used to amplify the V3–V4 regions (Klindworth et al., 2013). Amplification was performed using the Truseq (TS)-tailed1-step amplification protocol (Raju et al., 2018). Following PCR amplification, samples were pooled in equal volumes. The sample pool was analyzed on the Agilent 2100 Bioanalyzer using the Agilent High Sensitivity DNA Kit (Agilent Technologies Inc., Santa Clara, CA, United States) to quantify amplification performance and yield. The sequencing of PCR amplicons was performed using the Illumina HiSeq1500 instrument (Illumina, Inc., San Diego, CA, United States). Samples, together with nine blank (negative control) samples (Raju et al., 2018) and two water samples, were sequenced at 270 bp paired-end reads, providing sufficient overlap of high-quality sequences between the forward and reverse reads, thus reducing the error rates.

### Operational Taxonomic Units (Otus)

Sequencing quality and length filtering was carried out and sequences were processed using the MiSeq SOP in the mothur pipeline (Version v.1.35.1) (Schloss et al., 2009), also described in details by Raju et al. (2018). We used the SILVA 16S rRNA database (Version V119) and taxonomy for the alignment and classification since it provides comprehensive, quality-checked and regularly updated databases of aligned small (16S/18S, SSU) and large subunits (Quast et al., 2012). To ensure high-quality data for the analysis, sequence reads containing ambiguous bases, homopolymers >8 bp, more than one mismatch in the primer sequence, less than 10 basepair assembly overlap or sequences under the default per base quality score in mothur were removed. Assembled reads >460 bp in length and singletons were excluded from the analysis. This substantially reduced the number of assembled reads, ensuring high quality data. Chimeric sequences were also removed from the dataset using the UCHIME algorithm within the mothur pipeline (Edgar et al., 2011). The high-quality sequence reads were aligned to the Silva 16S rRNA database and clustered into OTUs at a cut-off value >98%. OTU was normalized by subsampling with threshold of 500 excluding the minimum number of samples (*n* = 52, inclusive 9 underweight, 38 normal weight, and 5 overweight), and rarefaction of data was performed. This approach was shown to produce highly reproducible microbiota profiles with very little contamination (Raju et al., 2018). Alpha diversity (Inverse Simpson index) was calculated per sample. Beta diversity (i.e., the variation in community composition between microbiota samples) using the Bray Curtis dissimilarity indices was calculated between the samples/body size, and gender. Sequencing depths were categorized into three groups: low <10,000, medium <100,000, high >100,000 sequences and used as a covariate in the statistical models.

### Statistics

Considering 100,000 sequences per sample, a minimum difference of 10% between groups and a significance level of 5% in diversity index, we have estimated the statistical power for this study with 1000 samples to be greater than 80% (Sze and Schloss, 2016). Kruskal–Wallis tests were performed to assess the significant distribution of alpha-diversity measures across the body size groups, and gender. We also tested the interactions between gender and body size in the model. Permutational analysis of variance (PERMANOVA) test with 999 permutations was performed using the function adonis and betadisper in Phyloseq R-package to test for differences in bacterial community structure among groups of samples. Differentially abundant OTUs were identified at the taxonomic levels of Order, Genus, and OTUs using the zero-inflated Gaussian model (*fitZig*), implemented in the metagenomeSeq package (Paulson et al., 2013; Paulson, 2014). Associations between: (a) normal weight vs. underweight, (b) normal weight vs. overweight, (c) normal weight vs. obese, (d) normal weight vs. overweight + obese, and (e) boy vs. girl were tested. Analyzes were adjusted for the sequence depth and gender, and the gender model was adjusted for sequence depth only. *P*-values were calculated and adjusted by the false discovery rate (FDR). Community types, similar to enterotypes, of the participants based on phylotype data were also checked (Arumugam et al., 2011).

## Results

We obtained saliva microbial data from 900 consented children, who were categorized into four body size groups (Table 1). Among the children, 96% were 11 or 12 years old at the time of sampling. In total, 73.3% of the children were of normal weight; there were no statistically significant gender differences in the proportion of overweight and obese children. However, there was a higher proportion of underweight girls than boys (15.9% vs. 11.5%).

### Deep Sequencing of the Saliva Microbiota

The 16S rRNA amplicons Illumina sequencing generated approximately 133 million raw read pairs and rarefaction analysis for all the saliva samples showed that the sequencing approach and depth was sufficient to determine the bacterial richness (Supplementary Figures S1a,b). The samples had a median read pair count of 113,345, mean read pair count of 148,508 and a maximum read pair count of 1.3 million. On average 48,248 read pairs were assembled per sample and these were assigned to 6536 OTUs. A few OTUs, especially OTU#12, were mostly assigned to negative control samples.

### Diversity of Children’s Saliva Microbiota

**FIGURE 1:**
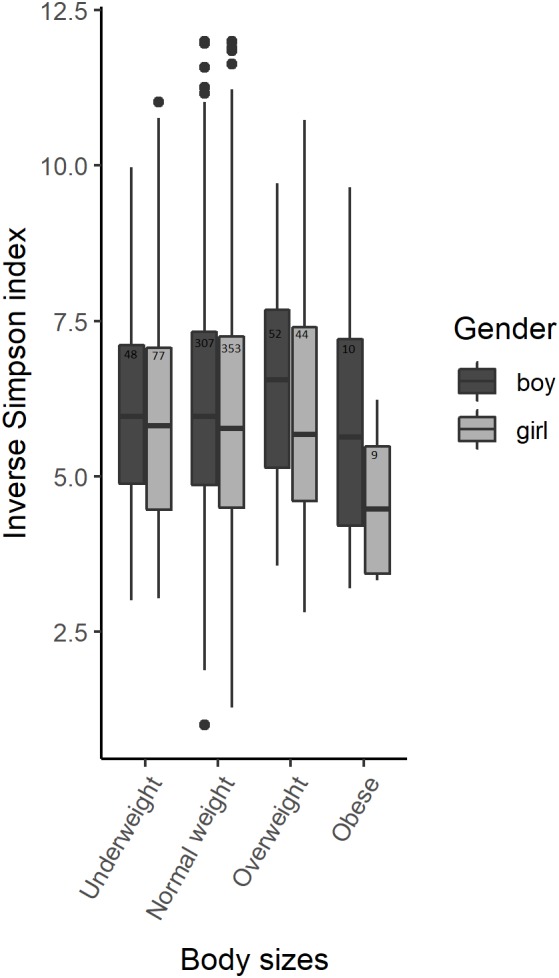
Alpha-diversity among body sizes in boys and girls saliva microbiota. Box plots illustrating the comparison of Inverse Simpson index between the saliva microbiota of boys and girls amongst 900 participants of the Finnish Health in Teens (Fin-HIT) cohort.

No statistically significant alpha-diversity differences were observed between body sizes when analyzing all samples using the Inverse Simpson index from a subsampled OTU dataset. However, alpha diversity was significantly different between boys and girls (Figure 1 and Supplementary Table S1). We identified statistically significant alpha diversity when comparing normal weight girls with overweight girls (*p*-value = 0.04) and normal weight boys with obese boys (*p*-value = 0.05) in gender-stratified analyses (Figure 1 and Supplementary Table S2). We did not find any significant interaction between alpha-diversity, body size and gender.

There was a statistically significant microbiota composition difference (beta diversity) between normal weight vs. obese children (*F*-value = 1.9736, *p*-value = 0.041) and between boy vs. girl (*F*-value = 4.421, *p*-value = 0.001) (Table 2). Beta diversity showed significant overall microbiota differences between normal weight girls vs. obese girls (*F*-value = 2.1921, *p*-value = 0.028) (Table 3) in the gender stratified analyses.

**Table 2:** Beta diversity compared among the body sizes of Finnish children.

	*R*^2^	*P*-value
Normal – underweight	0.0013	0.527
Normal – overweight	0.0012	0.545
Normal – overweight + obese	0.0017	0.251
Normal – obese	0.0032	0.041
**Gender**		
Boy – Girl	0.0054	0.001


*Results of PERMANOVA analysis for the beta diversity in body sizes and gender using Bray Curtis dissimilarity index amongst 900 participants of the Fin-HIT cohort.*

**Table 3:** Beta diversity of saliva microbiota among finnish boys and girls.

	Body sizes	*R*^2^	*P*-value
Boys	Normal – underweight	0.00225	0.707
	Normal – overweight	0.00167	0.891
	Normal – overweight+ obese	0.0047	0.742
	Normal – obese	0.00341	0.448
Girls	Normal – underweight	0.00245	0.536
	Normal – overweight	0.00245	0.536
	Normal – overweight+ obese	0.00839	0.063
	Normal – obese	0.00654	0.028


*Results of PERMANOVA analysis for the beta diversity in boys (*n* = 417) and girls (*n* = 483) and body sizes using Bray Curtis dissimilarity index amongst 900 participants of the Fin-HIT cohort.*

### Composition of Saliva Microbiota

We assigned 257 phylotypes and 6536 OTUs at 98% sequence similarity. From these data, we identified a total of 19 phyla and 135 genera. Fourteen bacterial genera (*Veillonella, Prevotella, Streptococcus, Neisseria, Selenomonas, Haemophilus, Eubacterium, Porphyromonas, Fusobacterium, Gemella, Campylobacter, Granulicatella, Leptotrichia*, and *Johnsonella*) were present in more than 95% of the samples, here defined as the core saliva microbiota. Firmicutes (51%), Bacteroidetes (20%), Proteobacteria (16%), Actinobacteria (6%), Candidate division TM7 (4%), and Fusobacteria (3%) were the 6 major phyla, accounting for 99.81% of saliva bacteria. The average number of OTUs per sample, reflecting microbial richness, was 227. There was no significant difference in the number of phylotypes between body sizes and gender. 16 abundant and 211 rare OTUs accounted for 86% and 14% of the assembled sequences, respectively (Supplementary Figure S2a) in all body sizes (Supplementary Figure S2b). The top five abundant OTUs present in the saliva samples belonged to the genera *Veillonella*, *Prevotella*, *Streptococcus*, *Selenomonas*, and *Neisseria*.

We also examined the ratio of Bacteroidetes to Firmicutes, which are known for their association with BMI (Ley et al., 2006), but no association was found when comparing normal weight to overweight or obese individuals. The predominant bacterial classes in the saliva microbiota of the children were Clostridia (39.4%), Bacteroidia (19.3%), Bacilli (11.8%), Betaproteobacteria (7.5%), Gammaproteobacteria (7.4%), Actinobacteria (6.3%), and unclassified bacteria (4.3%). Among all the predominant bacterial classes, Clostridia were highly dominant in all of the saliva samples (Supplementary Figure S3). Using an approach similar to that used to identify enterotypes in intestinal microbiota (Arumugam et al., 2011), we identified three clusters in the saliva microbiota (Supplementary Figure S4). The clusters are defined by differences in abundance. Cluster 1 and cluster 3 are more similar than the smaller cluster 2. There were no statistically significant associations between body sizes or gender and the three clusters.

### Differentially Abundant Microbes in Children With Different Body Sizes

**FIGURE 2:**
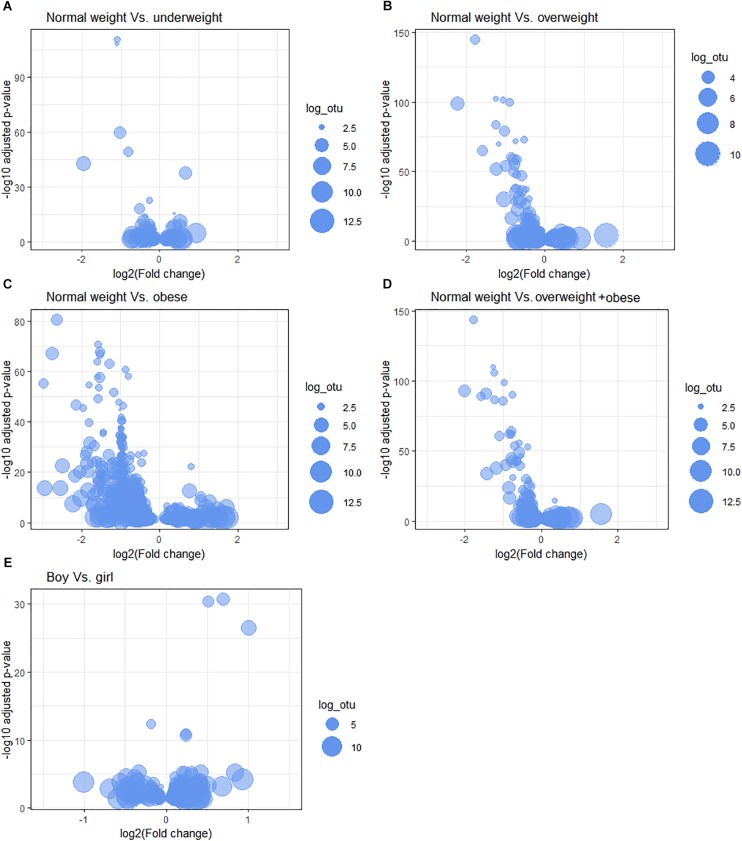
Microbiota relative abundance differences between body sizes. The following comparisons are shown: **(A)** normal weight vs. underweight, **(B)** normal weight vs. overweight, **(C)** normal weight vs. obese, **(D)** normal weight vs. overweight + obese, and among gender **(E)** boys vs. girls at OTU-taxonomic. Bubble size represents the log count of OTU (log_otu).

We tested the associations between the relative abundance of bacteria at different taxonomic levels and children’s body sizes; the results are represented by volcano plots and quantile-quantile (Q-Q) plots (Figure 2 and Supplementary Figure S5). We report the top five differentially abundant bacteria at each taxonomic level.

#### Normal Weight vs. Underweight

All of the top five OTUs were relatively less abundant in underweight children (adjusted *p*-value < 0.05) (Figure 2A and Table 4). The OTUs belonged to the genera *Selenomonas, Streptococcus, Haemophilus, Leptotrichia*, and family Bifidobacteriaceae. At the genus level, bacteria belonging to the Enteric Bacteria cluster, *Kingella*, and *Anaerovorax* showed significantly less relative abundance, and those related to *Lactococcus* and *Simonsiella* were significantly elevated in the underweight group (Supplementary Figure S6a and Supplementary Table S3A). At the order level, Enterobacteriales and Synergistales were significantly less abundant in the saliva of underweight children compared to the normal weight group (Supplementary Figure S6a and Supplementary Table S3B).

**Table 4:** Differentially abundant bacteria by children’s body sizes.

	OTU (nearest taxa)	log_2_FC	*P*-value	p-adj	Log odds
a	Otu001424_Selenomonas	–1.1	8.89E-115	1.63E-111	246.63
	Otu002086_Streptococcus	–1.09	5.06E-112	4.65E-109	242.95
	Otu000774_Haemophilus	–1.02	4.84E-63	2.97E-60	132.97
	Otu000991_Leptotrichia	–0.81	9.43E-53	4.33E-50	109.43
	Otu000494_Bifidobacteriaceae	–1.96	3.05E-46	1.12E-43	94.59
b	Otu001374_Neisseriaceae	–1.78	8.05E-149	1.48E-145	326.70
	Otu001829_*Neisseriaceae*	–1.25	8.43E-106	7.74E-103	230.76
	Otu001913_Micrococcineae	–1.07	9.60E-105	5.88E-102	228.48
	Otu001546_Catonella	–0.9	6.62E-103	3.04E-100	224.15
	Otu000822_*Neisseriaceae*	–2.23	8.90E-102	3.27E-99	221.64
c	Otu001013_Veillonella	–2.64	1.11E-84	2.04E-81	182.71
	Otu001241_Prevotella	–3.55	3.86E-76	3.55E-73	162.95
	Otu001965_Selenomonas	–1.58	3.26E-74	2.00E-71	158.42
	Otu000997_Selenomonas	–1.53	4.25E-71	1.95E-68	151.51
	Otu000573_Streptococcus	–2.77	1.86E-70	6.86E-68	150.02
d	Otu001374_Neisseriaceae	–1.78	7.86E-148	1.45E-144	324.93
	Otu001342_Prevotella	–1.27	5.72E-114	5.25E-111	249.33
	Otu001629_Candidate_division_TM7	–1.22	6.59E-109	4.04E-106	237.51
	Otu001878_Selenomonas	–0.98	2.74E-102	1.26E-99	222.86
	Otu000822_Neisseriaceae	–2.00	2.96E-96	1.09E-93	209.16
e	Otu000350_Neisseria	1.11	2.07E-33	3.81E-30	64.72
	Otu000616_Actinomycineae	0.69	9.72E-32	8.93E-29	60.29
	Otu000754_Streptococcus	0.46	4.48E-29	2.75E-26	54.25
	Otu000889_Haemophilus	0.37	5.46E-22	2.51E-19	38.32
	Otu000755_Oribacterium	0.26	2.01E-15	7.39E-13	24.49


*Differentially abundant bacteria at OTU-level for the body sizes; (a) normal weight vs. underweight, (b) normal weight vs. overweight, (c) normal weight vs. obese and (d) normal weight vs. overweight + obese, as well as (e) boys vs. girls amongst 900 participants of the Fin-HIT cohort.*

#### Normal Weight vs. Overweight

The top five OTUs were found to be relatively less abundant in the saliva of the overweight group. Three of these OTUs were assigned to unclassified Neisseriaceae, one to Micrococcineae bacteria and the last OTU was assigned to the genera *Catonella* (Figure 2B and Table 4). At the genus level, *Acinetobacter, Macrococcus, Alysiella, Acidovorax*, and *Bradyrhizobium* were significantly less abundant (Supplementary Figure S6b and Supplementary Table S3A) in overweight children. At the order level, Planctomycetales were comparatively lower and Acidobacteriales were higher in relative abundance levels in overweight children (Supplementary Figure S6b and Supplementary Table S3B).

#### Normal Weight vs. Obese

The top five OTUs were significantly less abundant in obese compared to normal weight children. The OTUs belonged to genera *Veillonella*, *Prevotella*, and *Streptococcus* and two OTUs to *Selenomonas* bacteria (Figure 2C and Table 4). Five genera, *Enhydrobacter*, *Bulleidia*, *Acinetobacter, Bergeriella*, and *Simonsiella*, were significantly less abundant in the saliva of obese children (Supplementary Figure S6c and Supplementary Table S3A). The orders Acidobacteriales, Mycoplasmatales, Caulobacterales, and Myxococcales were comparatively more abundant, whereas Coriobacteridae were one-fold lower in abundance in the saliva of obese children (Supplementary Figure S6c and Supplementary Table S3B).

#### Normal Weight vs. Overweight + Obese

The top five OTUs were significantly less abundant in all children with overweight and obese combined group when comparing to normal weight children. Two OTUs belonged to unclassified Neisseriaceae bacteria, one Candidate_division_TM7, and genera *Prevotella* and *Selenomonas* were less abundant in the saliva of children with overweight + obese (Figure 2D and Table 4). At the genus level, *Acinetobacter, Alysiella, Simonsiella, Xylanibacter*, and *Acidovorax* were up to 0.5 2-fold lower in the children with overweight + obese combined (Supplementary Figure S6d and Supplementary Table S3A). At the order level, only Acidobacteriales were comparatively low in the children with overweight + obese combined (Supplementary Figure S6d and Supplementary Table S3B).

#### Oral Microbiota in Boys and Girls

The top five OTUs that were significantly more abundant in girls were: *Neisseria*, Actinomycineae, *Streptococcus, Haemophilus*, and *Oribacterium* (Figure 2E and Table 4). At the genus level, *Brachymonas, Sphingomonas, Lactococcus*, *Mesorhizobium*, and *Paludibacter* were less abundant in girls (Supplementary Figure S6e and Supplementary Table S3A). Bacteria belonging to the orders Caulobacterales and Rhodospirillales were less abundant, whereas Mycoplasmatales were increased in girls (Supplementary Figure S6e and Supplementary Table S3B).

## Discussion

In this study, including 900 Finnish children, we observed an association between the saliva microbiota and body size. The differences in alpha and beta diversity and bacterial abundance between body sizes were gender-dependent, suggesting that the saliva microbial community–body size associations differ in boys and girls. However, no significant interactions were identified. To our knowledge, this is the first large-scale study that explores the association of the saliva microbiota composition and body sizes in children. Our sample size–a total of 483 girls and 417 boys–ensured statistical power to detect differences in microbiota abundance and alpha and beta diversity according to body size.

A recent study of the gut microbiota reported that distinctive bacteria were associated with men and women, and these differences between gender may be influenced by the severity of obesity (Haro et al., 2016), in line with our gender-specific microbiota differences between body sizes. The gender differences observed in saliva microbiota diversity may be induced by the endocrine system as our study participants are about to enter puberty, which may contribute to a shift in the oral microbiota (Zaura and Ten Cate, 2015).

We observed large log-odds ratios differences in microbial abundance between underweight, normal weight, overweight and obese children. A notable finding is the many-fold decrease in the relative abundance of core saliva bacteria in all body sizes, including the abundant genera *Veillonella, Prevotella, Selenomonas*, and *Streptococcus* (in obese children). Saccharolytic bacteria–*Streptococcus*, *Actinomyces*, and *Lactobacillus* species—degrade carbohydrates into organic acids, resulting in dental caries (Takahashi, 2015). Proteolytic/amino acid–degrading bacteria–*Prevotella* and Fusobacterial species–break down proteins and peptides to produce short-chain fatty acids, ammonia, sulfur compounds, and indole/skatole, which act as infectious and modifying factors in periodontitis and oral malodour (Takahashi, 2015). Our results showed that *Prevotella* is associated with body size, and may potentially be novel markers to identify those at risk of gaining excess weight. However, further studies are needed to replicate our finding and then investigate the potential causal pathways involved.

Several studies have shown that the gut microbiota is associated with body size and may influence host metabolism, release of gut hormones and energy uptake from the diet (John and Mullin, 2016). The body size related saliva bacteria identified in our study differs from the body size related intestinal bacteria identified by John and Mullin (2016), confirming the differences between oral and gut microbiota (Human Microbiome, and Project Consortium, 2012). *Veillonella, Prevotella, Streptococcus, Selenomonas*, *Neisseria, Haemophilus, Micrococcineae, Gemella*, and *Neisseria* were the abundant genera in the saliva of the children in our study, and these findings are in line with an earlier deep analysis of adult men (Lazarevic et al., 2012). A single species, *Selenomonas noxia*, has been used to classify the saliva of overweight women (Goodson et al., 2009). In our study, *Selenomonas* spp. was differentially less abundant in underweight, obese and overweight+obese compared to normal weight children, irrespective of gender.

The strengths of this study include the large sample size and homogeneous age group. In this particular age group, children do not normally suffer from periodontitis and chronic conditions, such as diabetes and cardiovascular diseases (Alabdulkarim et al., 2005; Sarlati et al., 2008). The limitation of this study include the lack of oral health status and puberty status of children, which are potential confounders. Our questionnaire included questions on puberty status, missing data and suspicion of reporting biases for this particular topic in this age group made us reluctant to include this information. In Finland most of the children and adolescents have good oral health, and public oral healthcare services are freely available for individuals under 18 years of age. Individual preventive efforts are provided for certain age groups and declining trends in the caries occurrence rate has been seen (Mattila et al., 2016). Moreover, in Finland, 11 and 12 year old children’s are unlikely to be dieting, smoking and consuming alcohol, which are factors that could influence our results. The exception might be underweight children, with higher prevalence in girls, and may be attributed to eating disorders. Previous studies have reported that compared to the gut microbiota, the saliva microbiota is more resilient and stable, despite changes in diet and oral hygiene (Stahringer et al., 2012). Thus, the saliva-based screening of microbial biomarkers, which are associated with oral and general health status, may be considered a feasible, simple, economical and non-invasive alternative (Schafer et al., 2014). We assume that a major part of the diet is at least partly similar in this age group and did not take into account the potential dietary confounding in this study. Children in Finland receive healthy lunches and snacks provided by the school system as part of their free education ^2^. The intake of fresh fruits, vegetables and legumes has been associated with lower rates of obesity, while a high intake of fats and sweets has been associated with obesity (Vilela et al., 2014; Tucker et al., 2015). Additional studies are needed to identify the contribution of diet to the saliva microbiota – body size association. Due to the young age of our study participants and the healthy school lunch, our findings may not be generalizable to adult and elderly populations, especially considering that the oral microbiota changes as a function of smoking and alcohol consumption.

## Conclusion

We observed that the saliva microbiota composition and abundance were significantly associated with body size and dependent on gender; particularly notable was the decrease in the core bacteria in overweight and obese children. In some respect, this mimics the findings in intestinal microbiota that are found to be associated with obesity, but the detailed mechanisms are waiting to be uncovered (John and Mullin, 2016). Overweight and obese children are likely to stay obese into adulthood and develop diseases more frequently than normal weight children. Thus, the early identification of subjects at risk of developing obesity and the prevention of overweight and obesity is of great importance. The associations identified here can direct future studies, such as interventions, to investigate the role of saliva microbiota in weight development and management in children.

## Ethics Statement

This study was carried out in accordance with the recommendations of regional Ethics Committee of the Hospital District of Helsinki and Uusimaa with written informed consent from all subjects. All subjects gave written informed consent in accordance with the Declaration of Helsinki. The protocol was approved by the regional Ethics Committee of the Hospital District of Helsinki and Uusimaa (169/13/03/00/10).

## Author Contributions

TR and EW designed the research project. SL, PE, and SR conducted the research. SR and TR analyzed the data. SR, TR, and WV interpreted the data. SR and TR wrote the manuscript with significant input from all other authors. All authors read and approved the final manuscript.

## Disclaimer

Since 1 January 2019, EW has been a staff member of the International Agency for Research on Cancer. Where authors are identified as personnel of the International Agency for Research on Cancer/World Health Organization, the authors alone are responsible for the views expressed in this article and they do not necessarily represent the decisions, policy or views of the International Agency for Research on Cancer/World Health Organization.

## Conflict of Interest Statement

The authors declare that the research was conducted in the absence of any commercial or financial relationships that could be construed as a potential conflict of interest.

## Footnotes

1 http://www.finhit.fi/

2 www.oph.fi
