# Supplementary material for: Gender-Specific Associations Between Saliva Microbiota and Body Size
Source: Front Microbiol. 2019 Apr 10;10:767. doi: 10.3389/fmicb.2019.00767 (PMC6467948; doi:10.3389/fmicb.2019.00767)
Supplement: Supplementary file 1 [file Table_1.docx]

**Supplementary Figure S1 a:** Histogram showing the distribution of 16S rDNA raw sequences sorted (Y-axis) in saliva samples (X-axis).


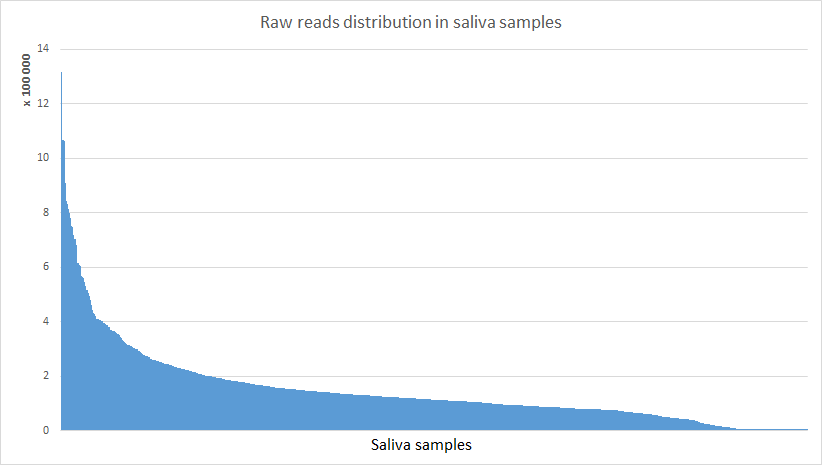


**Supplementary Figure S1 b:** Rarefaction curve showing the number of species vs number of sequences.


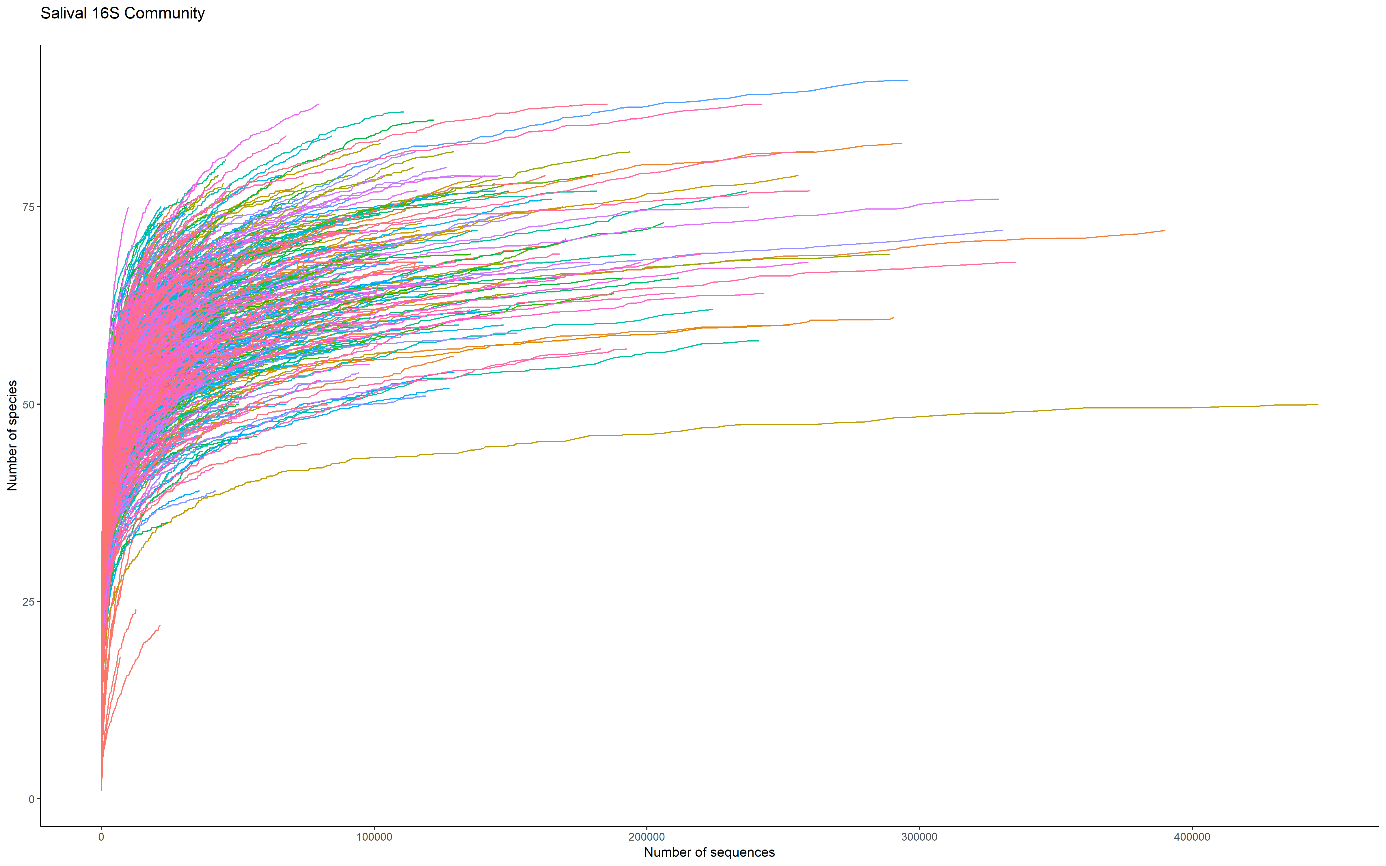


**Supplementary Table S1:** Kruskal–Wallis test for Inverse Simpson diversity indices by body size, and between genders.

| **Body sizes*** | **Inverse Simpson index** | | |
| --- | --- | --- | --- |
|  | **chi-square** | **df** | **p-value** |
| Normal- – underweight | 0.009 | 1 | 0.923 |
| Normal- – overweight | 1.379 | 1 | 0.240 |
| Normal weight – obese | 3.762 | 1 | 0.052 |
| Normal- – overweight + obese | 5.395 | 2 | 0.067 |
| Boy – girl | 6.112 | 1 | 0.013 |

**Supplementary Table S2:** Diversity of saliva microbiota compared among Finnish girls, and boys

|  | **Body sizes** | **Inverse Simpson index** | | |
| --- | --- | --- | --- | --- |
|  |  | **Chi-Square** | **df** | **p-value** |
| Girls | Normal - underweight | 6.39 | 3 | 0.09 |
|  | Normal - overweight | 8.15 | 3 | 0.04 |
|  | Normal weight - obese | 3.90 | 3 | 0.27 |
|  | Normal - overweight +obese | 5.62 | 3 | 0.13 |
| Boys | Normal - underweight | 3.73 | 3 | 0.29 |
|  | Normal - overweight | 1.59 | 3 | 0.66 |
|  | Normal weight - obese | 7.80 | 3 | 0.05 |
|  | Normal - overweight +obese | 5.57 | 3 | 0.13 |

Kruskal-Wallis (KW) test of Inverse Simpson diversity in girls’ and boys’ BMI categories amongst 900 participants of the Finnish Health in Teens - Fin-HIT - cohort.

**Supplementary Figure S2:**

a) Distribution of abundant and rare OTUs in the saliva samples

**
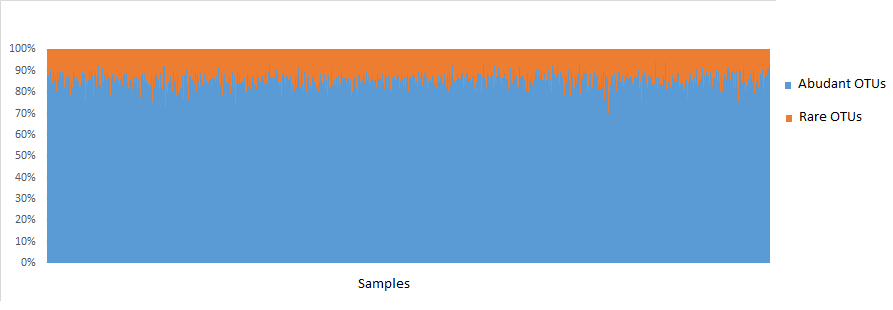
**

b) Relative distribution of abundant and rare OTUs in the underweight, normal weight, overweight and obese children

**
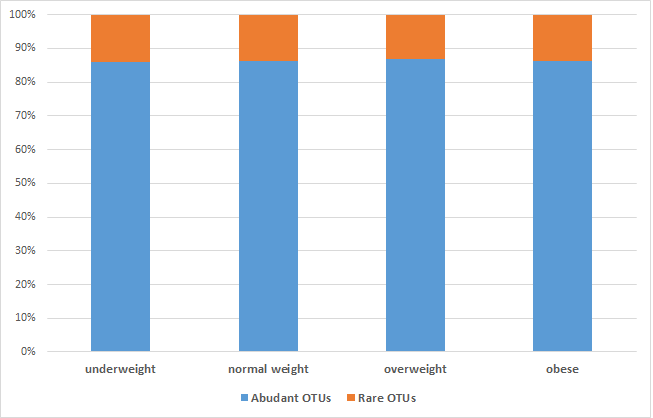
**

**Supplementary Figure S3**:

**Composition of major bacterial taxa.**

Stacked area plots show the abundant bacterial classes in children belonging to the four categories of body sizes (underweight; normal weight; overweight and obese, classified according to Cole and Lobstein, 2012); bars indicate relative abundances, coloured by taxonomic level class in boys (top) and girls (bottom).

**
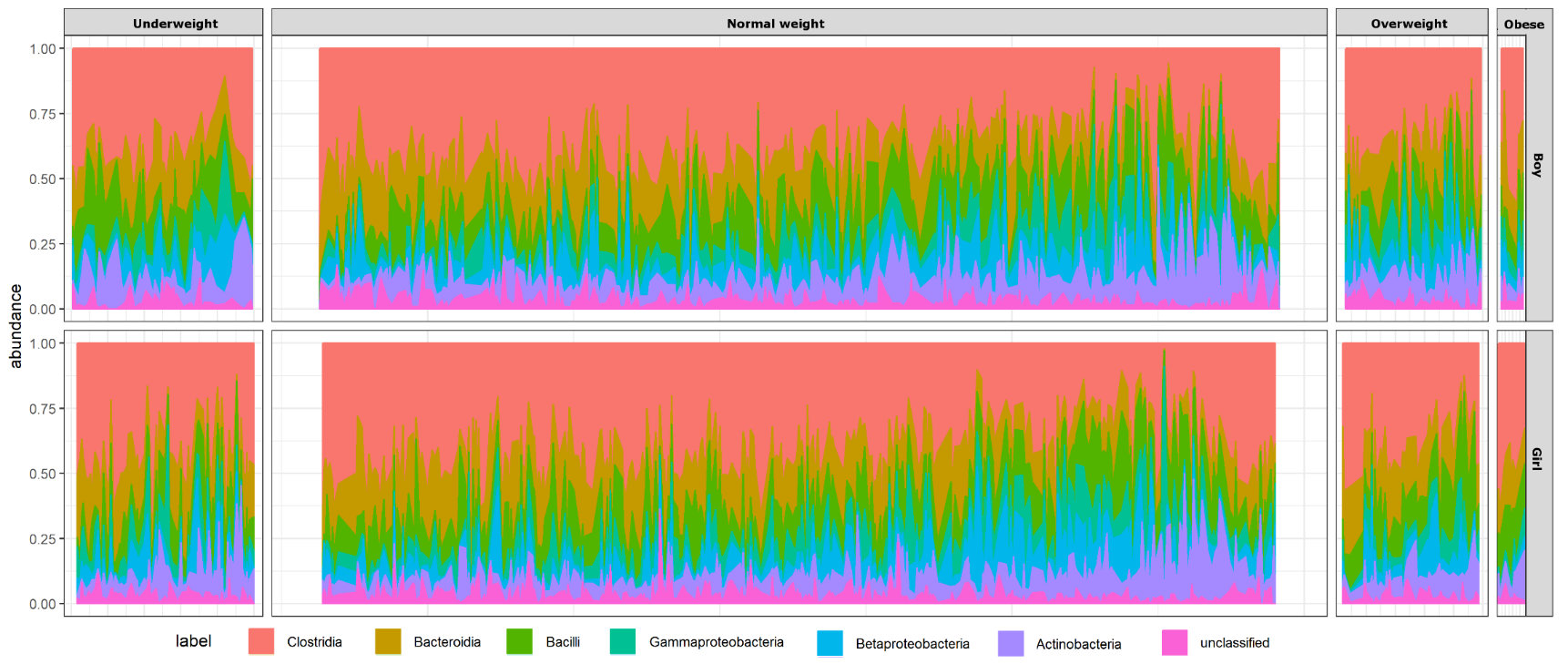
**

**Supplementary Figure S4**:

**Clustering of the microbiota of saliva samples.**

Finnish children’s saliva phylotype data were clustered using Jensen-Shannon divergence (JSD) and Partitioning Around Medoids (PAM) clustering. Three clusters was chosen by the Calinski–Harabasz index and were validated based on the prediction strength (PS) and average silhouette width (SW). Clusters shown as double Principle Coordinate Analysis (dPCOA) plot.

**
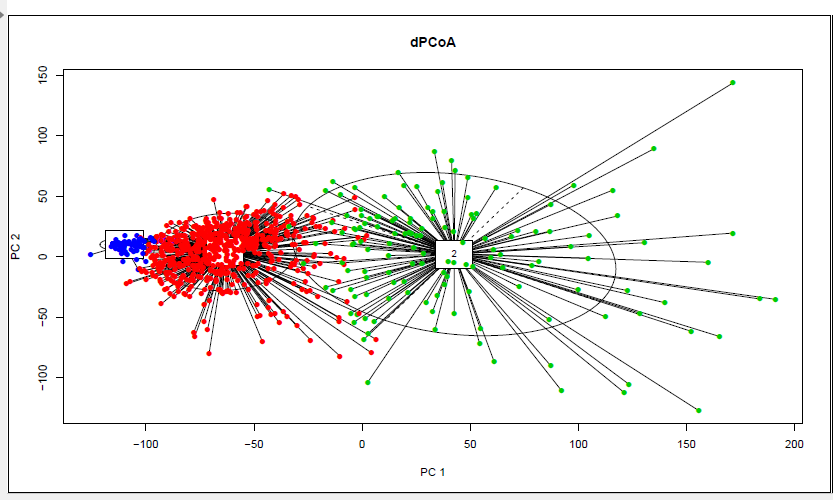
**

**Supplementary Figure S5**

Quantile–quantile (QQ) plots of expected (x-axis) and observed (y-axis) p-values of models used in this analysis where groups of body sizes: a) normal weight vs. underweight, b) normal weight vs. overweight, c) normal weight vs. obese and d) normal weight vs. overweight + obese, as well as e) boys vs. girls, saliva microbiota taxa classified at the OTU, genus and order level. P-values distantly diverged from the null line for different microbial groups (OTU, genus & order).

**
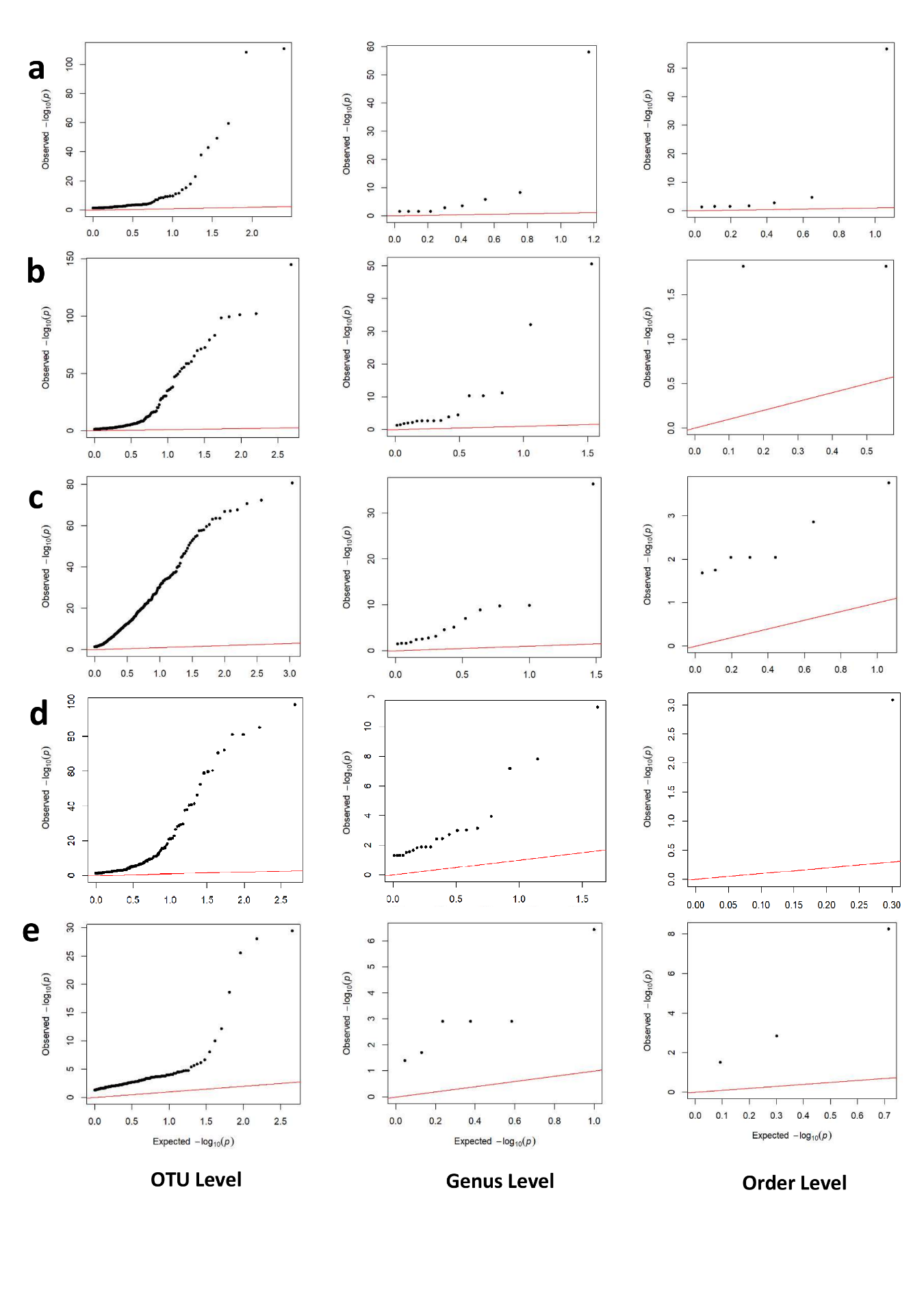
**

**Supplementary Figure S6. Microbiota abundance differences between body sizes.**

The following comparisons are shown: a) normal weight vs. underweight, b) normal weight vs. overweight, c) normal weight vs. obese, d) normal weight vs. overweight + obese and among gender e) boys vs. girls at taxonomic levels genus (red) and order (green). Bubble size is represented with the log count of taxa (i.e., genus and order).


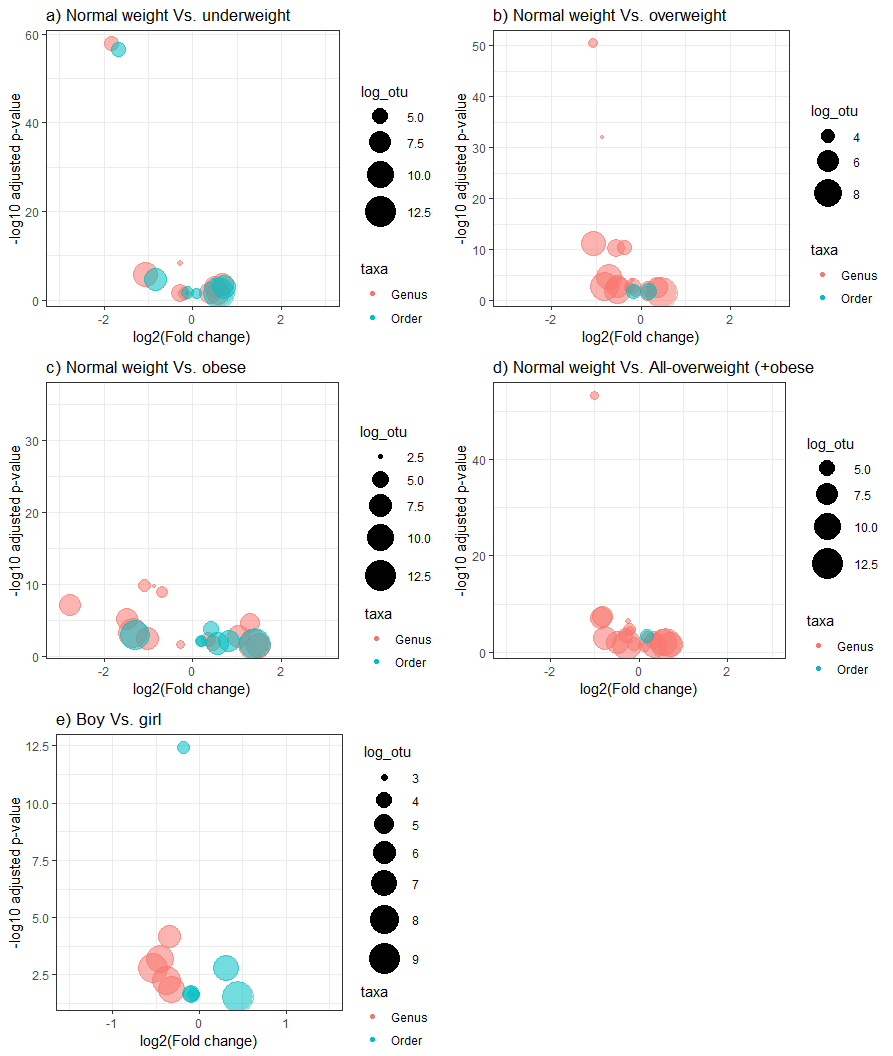


**Supplementary Table S3:** Differentially abundant bacteria by children’s body sizes at genus and order level.

**A)**

|  | **Taxa** | **log2FC** | **P.Value** | **p-adj** | **Log odds** |
| --- | --- | --- | --- | --- | --- |
| **a** | Enteric_Bacteria_cluster | -1.83 | 1.09E-60 | 9.94E-59 | 127.70 |
|  | Kingella | -0.27 | 1.09E-10 | 4.95E-09 | 12.99 |
|  | Anaerovorax | -1.07 | 5.25E-08 | 1.59E-06 | 6.59 |
|  | Lactococcus | 0.66 | 1.17E-05 | 0.0002 | 1.04 |
|  | Simonsiella | 0.52 | 7.84E-05 | 0.001426 | -0.68 |
| **b** | Acinetobacter | -1.06 | 3.20E-53 | 2.91E-51 | 110.53 |
|  | Macrococcus | -0.85 | 2.08E-34 | 9.47E-33 | 67.33 |
|  | Alysiella | -1.06 | 2.07E-13 | 6.28E-12 | 18.54 |
|  | Acidovorax | -0.37 | 2.04E-12 | 4.65E-11 | 16.62 |
|  | Bradyrhizobium | -0.55 | 2.65E-12 | 4.83E-11 | 16.33 |
| **c** | Enhydrobacter | -3.47 | 5.04E-39 | 4.58E-37 | 77.93 |
|  | Bulleidia | -1.09 | 3.10E-12 | 1.41E-10 | 17.24 |
|  | Acinetobacter | -0.87 | 6.22E-12 | 1.89E-10 | 16.58 |
|  | Bergeriella | -0.67 | 5.51E-11 | 1.25E-09 | 14.10 |
|  | Simonsiella | -2.76 | 5.11E-09 | 9.30E-08 | 9.70 |
| **d** | Acinetobacter | 1.0 | 5.39E-56 | 4.91E-54 | 116.89 |
|  | Alysiella | 0.83 | 6.58E-10 | 2.99E-08 | 10.61 |
|  | Simonsiella | 0.85 | 2.51E-09 | 7.61E-08 | 9.34 |
|  | Xylanibacter | 0.24 | 1.47E-08 | 3.34E-07 | 8.10 |
|  | Acidovorax | 0.23 | 1.23E-06 | 2.24E-05 | 3.58 |
| **e** | Brachymonas | -0.41 | 4.08E-09 | 3.72E-07 | 10.38 |
|  | Sphingomonas | -0.55 | 3.62E-05 | 0.001 | 1.51 |
|  | Lactococcus | -0.43 | 4.36E-05 | 0.001 | 1.31 |
|  | Mesorhizobium | -0.43 | 5.44E-05 | 0.001 | 1.25 |
|  | Paludibacter | -0.33 | 0.001 | 0.02 | -1.93 |

**B)**

|  | **Taxa** | **log2FC** | **P.Value** | **p-adj** | **Log odds** |
| --- | --- | --- | --- | --- | --- |
| **a** | Enterobacteriales | -1.69 | 6.51E-59 | 2.15E-57 | 123.58 |
|  | Synergistales | -0.85 | 1.09E-06 | 1.79E-05 | 3.67 |
|  | Sphingomonadales | 0.72 | 0.0001 | 0.002 | -1.33 |
|  | Planctomycetales | -0.13 | 0.0020 | 0.017 | -3.38 |
|  | Thermaceae | -0.11 | 0.0045 | 0.029 | -4.15 |
| **b** | Planctomycetales | -0.16 | 0.0005 | 0.015 | -0.40 |
|  | Acidobacteriales | 0.16 | 0.0009 | 0.015 | -1.01 |
| **c** | Acidobacteriales | 0.43 | 5.28E-06 | 0.0001 | 3.68 |
|  | Coriobacteridae | -1.29 | 8.36E-05 | 0.001 | 0.61 |
|  | Mycoplasmatales | 0.84 | 0.001 | 0.009 | -1.30 |
|  | Caulobacterales | 0.21 | 0.001 | 0.009 | -1.31 |
|  | Myxococcales | 0.21 | 0.001 | 0.009 | -1.36 |
| **d** | Acidobacteriales | -0.19 | 1.34E-05 | 0.000443 | 2.83 |
| **e** | Caulobacterales | -0.15 | 1.69E-10 | 5.58E-09 | 13.43 |
|  | Mycoplasmatales | 0.31 | 8.64E-05 | 0.001 | 0.60 |
|  | Rhodospirillales | -0.07 | 0.003 | 0.031 | -2.31 |

**Legend:** Differentially abundant bacteria at (A) genus and (B) order level for the body sizes; a) normal weight vs. underweight, b) normal weight vs. overweight, c) normal weight vs. obese and d) normal weight vs. overweight + obese, as well as e) boys vs. girls amongst 900 participants of the Finnish Health in Teens (Fin-HIT) cohort
